# The Automatic Emotion Regulation of Children Aged 8–12: An ERP Study

**DOI:** 10.3389/fnbeh.2022.921802

**Published:** 2022-06-21

**Authors:** Fang Liu, Chao Gao, Heming Gao, Wen Liu

**Affiliations:** ^1^College of Psychology, Liaoning Normal University, Dalian, China; ^2^Department and Institute of Psychology, Ningbo University, Ningbo, China

**Keywords:** automatic emotion regulation, children, Nogo-P3, temperament, social anxiety

## Abstract

Emotion regulation in childhood and adolescence is related to their social development. Better emotion regulation is associated with great individual academic performance and mental health. However, compared with the research on emotion regulation strategies, children’s automatic emotion regulation has been less investigated. Using event-related potential (ERP) technology, this study adopts the cued-emotion Go/Nogo paradigm to investigate the processing characteristics of automatic emotion regulation in children aged 8–12 years. The current study selected 34 younger group [16 boys, 18 girls, mean (M) ± SD = 8.91 ± 0.75], and 31 older group [18 boys, 13 girls, M ± SD = 11.26 ± 0.45]. The results showed that, for Nogo trials, the amplitude of N2 and P3 evoked by emotional faces were significantly larger than those evoked by neutral faces, reflecting the cognitive conflict experienced and the process of children’s automatic response inhibition to emotional stimuli, respectively. However, no significant difference in N2 and P3 amplitude were found in Go trials, which may indicate that children aged 8–12 showed similar top-down control and similar motivated attention in this experiment, respectively. Further analysis found that the negative affect of temperament was significantly positively correlated with Nogo-P3 induced by neutral pictures (*r* = 0.37, *p* < 0.001), and preadolescents’ social anxiety was significantly positively correlated with Nogo-P3 followed by neutral pictures (*r* = 0.31, *p* < 0.01). These findings can provide inspiration and empirical support for the promotion and intervention of emotion regulation in children and adolescents.

## Introduction

Emotion regulation refers to an internal and external process by which individuals monitor, evaluate, and modify their emotional responses (Gross, 1998). A large number of studies have pointed out that the ability of emotion regulation is closely related to children’s academic performance, positive social-emotional development, and mental health (Gross and John, 2003; Tugade and Fredrickson, 2007; Cole et al., 2009; Mega et al., 2014; Shapero et al., 2016). Some development studies indicate that the emotion regulation ability increases with age (DeCicco et al., 2012; Zimmermann and Iwanski, 2014). Emotion regulation comes in many forms, and most emotion regulation efforts aim to reduce negative emotions and enhance positive emotions to promote good mental health (Aldao et al., 2010; Gross, 2013).

According to whether consciousness participates in the process of regulating emotion, emotion regulation can be divided into intentional emotion regulation (IER) and automatic emotion regulation (AER), which include all aspects of the emotion generation process (Gross and Thompson, 2007; Gyurak et al., 2011; Braunstein et al., 2017). Compared with IER, AER does not require individual mental effort, with fewer attention resources, and can also reduce the subjective experience or physiological reactions of negative emotion (Mauss et al., 2007a,b; Williams et al., 2009; Gao et al., 2018; Chen et al., 2020). AER is a type of emotion regulation that is common in daily life and important to mental health. For instance, inadequate emotion regulation is a core feature of social anxiety (Mennin et al., 2009; Farmer and Kashdan, 2012; Helbig-Lang et al., 2015; Sheppes et al., 2015). The cued-emotion Go/Nogo paradigm is a widely used paradigm for investigating AER, which is designed based on the classical Go/Nogo paradigm (Zhang and Lu, 2012; Zhang et al., 2016). In the classical Go/Nogo paradigm, the amplitudes of frontal Nogo-N2 and NoGo-P3 were significantly greater than those of Go-N2 and Go-P3 (Lamm et al., 2006; Albert et al., 2010). In the emotional Go/Nogo task, emotion activates some of the same brain regions with response inhibition, including the anterior cingulate gyrus (ACC) (Goldstein et al., 2007; Berkman et al., 2009). Albert et al. (2011) suggested that Nogo-P3 amplitude was associated with both response inhibition and AER. Meanwhile, emotional faces induce greater Nogo-P3 amplitude and shorter latency in Nogo conditions than neutral faces (Zhang and Lu, 2012; Zhang et al., 2016). Therefore, Nogo-P3 may be an excellent indicator of AER. Zhang et al. (2016) found that emotional valence did not modulate the amplitude of Nogo-N2 (as shown in Zhang and Lu, 2012). However, in the Go trial, the Go-N2 amplitude evoked by emotional pictures was significantly greater than that evoked by neutral pictures (Zhang and Lu, 2012; Zhang et al., 2016). This evidence may indicate that Go-N2 is not associated with AER, but is an indicator of the level of attention resources recruited for response inhibition (Pessoa et al., 2002). However, a large number of studies are based on adult participants (Phillips et al., 2008; Zhang and Lu, 2012), and only a few studies have investigated the AER of adolescents (Zhang et al., 2016). To our knowledge, few studies explore the AER of children.

Temperament is an individual difference in emotion, attention, reactivity, and self-control, and this individual difference has a physiological basis (Zentner and Bates, 2008). The researchers pointed out that some dimensions of temperament form the basis for the development of emotion regulation in children, and temperament can explain the stability characteristics of emotion regulation ability across time and situation (Gross and John, 2003; Rothbart and Sheese, 2007). For example, Rueda and Rothbart (2009) suggested that children with higher levels of effort control were more likely to adopt adaptive emotion regulation strategies, while children with higher levels of negative emotion were more likely to adopt maladaptive emotion regulation strategies. In addition, Gross and John (2003) also pointed out that individual temperament differences reflecting negative emotion will affect the development of individual emotion regulation ability. Taken together, temperament plays an important role in intentional emotion regulation. However, whether AER is affected by temperament is still unclear.

The present study mainly focused on the AER of children. Given the event-related potentials (ERP) technology has been widely used to reflect underlying temporal mechanisms, this study adopted the cued-emotion Go/Nogo paradigm combine with ERP to investigate the temporal mechanisms of AER. Since emotional stimulation will automatically capture individual attention and maintain the state of attention to promote the completion of tasks (Eimer and Holmes, 2007; Bayle and Taylor, 2010), we supposed the first hypothesis.

H1: Compared with Go-N2 evoked by neutral stimulation, emotional stimulation will induce a smaller Go-N2.

Subsequently, according to previous studies (Goldstein et al., 2007; Berkman et al., 2009; Albert et al., 2011), AER interacts with response inhibition and activates similar brain regions, which may lead to greater response inhibition, we supposed the second hypothesis.

H2: Compared with Nogo-P3 induced by neutral stimulation, the Nogo-P3 elicited by emotional stimulation might be greater.

## Materials and Methods

### Participants

A total number of 75 children participated in the current study. Seven children were excluded from data processing due to too many artifacts in electroencephalogram (EEG) data. Therefore, the data of 65 subjects were finally included in the final data analysis]8–12 years old, mean (*M*) ± *SD* = 10.031 ± 1.334]. All subjects had normal vision or corrected vision, and were in good physical and mental condition. According to the age and grade range of the subjects, the subjects were divided into two groups: the middle-grade group, children in grades 3–4 (*M* ± *SD* = 8.912 ± 0.754, 16 boys); and the senior grade group, children in grades 5–6 (*M* ± *SD* = 11.258 ± 0.445, 13 girls). Written informed consent was obtained from all the children, their parents, and affiliated school.

### Stimulus Materials and Psychological Measure

A total number of 10 neutral, positive, and negative faces were selected from the Chinese face affective picture system (CFAPS; Wang and Luo, 2005), and 10 children (not participating in the formal experiment) were recruited to score the arousal and valence of the selected 30 face pictures. The results showed that the face type had significant main effects on the emotion valence (*p* < 0.001), and the main effect on emotion arousal was not significant (*p* > 0.05).

#### Social Anxiety Scale for Children

The Social Anxiety Scale For Children (SASC) was administered to measure the severity of child social anxiety, which was developed by La Greca and Stone (1993) and consisted of 10 items rated from 0 (no such problem) to 2 (a problem often occurs). The higher score represented severe social anxiety symptoms. In this study, the internal consistency of the questionnaire was 0.855.

#### Early Adolescent Temperament Questionnaire-Revised

The Early Adolescent Temperament Questionnaire-Revised (EATQ-R) was administered to measure the severity of child temperament, which was developed by Ellis and Rothbart (2001) and consisted of 65 items rated from 1 (almost never) to 5 (almost always). This study selected two subscales: effort control and negative affect. The effort control subscale contains 16 items. A high score represents a high level of effort and control. A total of 13 items were included in the negative affect subscale. The higher the score, the higher the negative effect level. The internal consistency of the two subscales is 0.823 and 0.785, respectively.

### Design

Participants performed a cued-emotion Go/Nogo task with a 1:2 proportion of Go and Nogo trials. According to the paradigm of Zhang and Lu (2012), a 2 (age group: younger children and older children) × 2 (trial category: go and nogo) × 3 (emotion face type: neutral, negative, and positive) design was adopted, with the age group as the between-subject variable. The Chinese characters “男 (male)” and “女 (female)” were adopted to be the cue, and the emotion type was represented by the emotion face. Participants judged the gender of the emotion face according to the preceding cue. If the gender of the emotion face is consistent with the cue, participants should press “SPACE” on the keyboard as soon as possible (Go trials); if the gender of the emotion face was not matched with the cue, participants should only view the emotion face and did not press any button (Nogo trials). Throughout the experiment, participants were required to ignore the emotion of the face but focus on the gender of the face. E-prime 2.0 (Psychology Software Tools Inc., Pittsburgh, PA, United States) was used for both stimulus presentation and recording responses.

### Procedure

**FIGURE 1:**
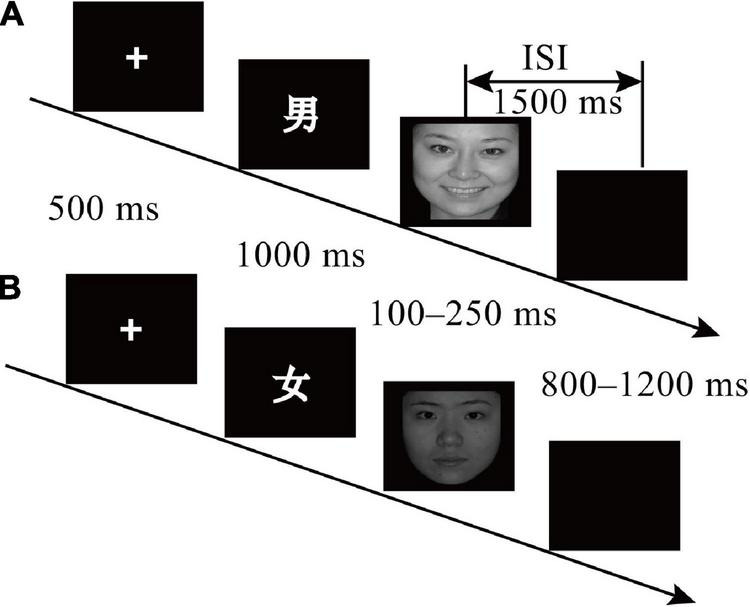
Schematic representation of the experimental procedure. **(A)** Nogo trial and **(B)** Go trial. Facial images sourced from CFAPS database (Wang and Luo, 2005). Reproduced with permission.

Each trial began with a “+” for 500 ms, then the cue was present for participants 1,000 ms. Followed by the emotion face randomly presented between 100 and 250 ms, and on this screen, participants should press the “SPACE” in the Go trial but not press any button in the Nogo trial. The screen then went blank for a random period ranging between 800 and 1,200 ms before the next trial began (Figure 1).

This experiment consisted of 324 trials split into 27 randomized blocks. Each block covered 12 trials. Participants were allowed to take a break every 9 blocks. The first three blocks were used as practice and were excluded from the data analysis.

### Behavior Data Analysis

The repeated-measure ANOVAs with the age group (younger children and older children) as a between-subject factor, and emotion face type (neutral, negative, and positive) as a within-subject factor were performed on the Go-, Nogo-accuracy, and Go-reaction time (RT).

### EEG Recordings and Analysis

The EEG was recorded from 64 Ag/AgCl electrodes mounted in an elastic cap with reference electrodes placed on the CPz site (ANT Inc., Germany). The electrode positions were according to the extended 10–20 system (Nuwer et al., 1998), and the electrooculogram (EOG) was recorded from a vertical facial electrode (below the left eye) and a horizontal facial electrode (on the right side of the right eye). The EEG data were sampled at 500 Hz/channel with a 0.1–100 Hz band-pass, and before the recording, all electrode impedances were maintained below 10 kΩ.

Raw EEG data were processed offline using the Brain Vision Analyzer (2.0, Brain Products GmbH). The raw data were first re-referenced to the average values at the left and right mastoids. Then, the EEG signals were digitally filtered using a 30 Hz low-pass filter. Followed by ocular correction using independent component analysis (ICA). The EEGs were segmented into 1,200 ms epochs surrounding the onset of the emotion face, and baseline-corrected with respect to the 200 ms pre-stimulus. Trials contaminated with electrooculogram artifacts or those with artifacts due to amplifier clipping, bursts of electromyographic activity, or peak-to-peak deflection exceeding ±80 μV were excluded from averaging. EEGs recorded from the different conditions were averaged separately for each participant, and only trials with correct responses were included in ERP averages.

For ERP elicited by the emotion face, the N2 (200–350 ms) and P3 (350–700 ms) time windows were selected for statistical analysis. The time periods corresponded to the typical latency ranges of the N2 and P3 components, respectively (Zhang and Lu, 2012; Zhang et al., 2016). Based on the previous studies and grand average, 9 electrodes were selected for statistical analysis. These electrodes were placed over three scalp regions (frontal: F3, Fz, and F4; frontal-central: FC4, FCz, and FC4; and central: C3, Cz, and C4). Repeated-measure ANOVAs with class level (middle and senior) as between-subject factor, and with trial category (Go and Nogo), emotion face type (neutral, negative, and positive) as within-subject factors were performed on the mean amplitudes of the N2 and P3 components.

Statistical calculations were carried out with IBM SPSS Statistics for Windows (IBM cooperation, Armonk, NY, United States). All effects with more than one degree of freedom were adjusted for sphericity violations using the Greenhouse–Geisser correction. The main effects were followed by the Bonferroni-corrected pairwise comparisons.

## Results

### Behavior Results

For the RT of Go trials, ANOVA revealed a significant main effect of emotion face type, *F*_(2,126)_ = 14.496, *p* < 0.001, η*_*p*_*^2^ = 0.187, indicating a faster RT for negative face (489 ms) and positive face (489 ms) than neutral face (5 than 10 ms), *p*s < 0.001, but no difference between negative and positive face was found (*p* = 1.000). The main effect of the age group was not significant, *F*_(1,63)_ = 1.130, *p* = 0.292; and the interaction between age group and emotion face type was not significant, *F*_(2,126)_ = 0.593, *p* = 0.554.

For the accuracy of Go trials, a significant main effect of age group was observed, *F*_(1,63)_ = 5.693, *p* = 0.02, η*_*p*_*^2^ = 0.083, with higher accuracy for the older group (90.5%) than the younger group (87.2%). The main effect of emotion face type did reach significance, *F*_(2,126)_ = 23.172, *p* < 0.001, η*_*p*_*^2^ = 0.269, indicating a higher accuracy for positive (90.7%) than neutral face (86.2%), *p* < 0.001, and for negative (89.7%) than neutral face (*p* < 0.001), but the difference between positive face and negative face was not significant (*p* = 1.000). The interaction between age group and emotion face type was not significant, *F*_(2,126)_ = 0.245, *p* = 0.783.

**FIGURE 2:**
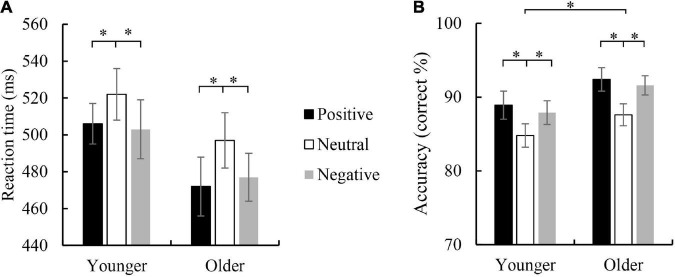
Mean reaction time **(A)** and accuracy **(B)** were elicited by viewing positive, neutral, and negative faces in the correct Go trials in younger and older children. Error bars denote the standard error (SE). *p < 0.05.

For the accuracy of Nogo trials, the main effect of age group did not reach significance, *F*_(1,63)_ = 0.283, *p* = 0.283. A significant main effect of emotion face type was observed, *F*_(2,126)_ = 28.734, *p* < 0.001, η*_*p*_*^2^ = 0.313, indicating the accuracy for positive (77.7%) and negative (76.3%) face were higher than neutral face (68.9%), *p*s < 0.001, but the difference between positive and negative was not significant (*p* = 0.761). The interaction between age group and emotion face type was not significant, *F*_(2,126)_ = 0.282, *p* = 0.755. Behavioral RT and accuracy are shown in Figure 2.

### ERP Results

**FIGURE 3:**
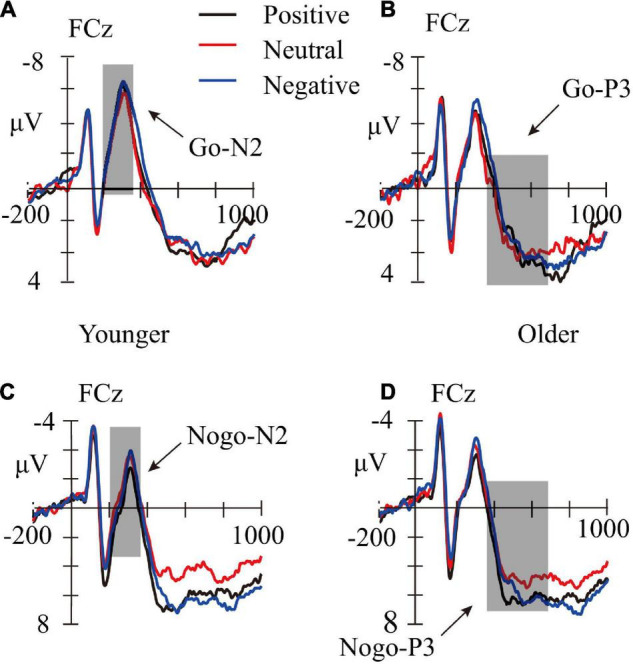
Averaged waveforms (μV) for positive (black lines), neutral (red lines), and negative pictures (blue lines) in Go-Younger **(A)**, Go-Older **(B)**, Nogo-Younger **(C)**, and Nogo-Older **(D)** conditions. The shadows represent time windows for N2 (200–350 ms) and P3 (350–700 ms).

**FIGURE 4:**
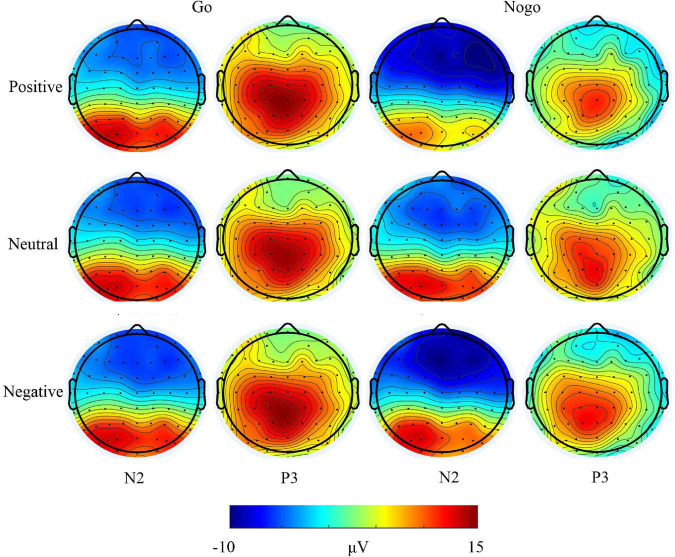
Topographic maps of all participants in different conditions.

A repeated-measure ANOVAs with age group (younger children and older children) as a between-subject factor, and with trial category (Go and Nogo), emotion face type (neutral, negative, and positive) as within-subject factors were performed on the mean amplitudes of the N2 and P3 components. The FCz site was selected for statistical analysis, as the main effect of electrodes showed that N2 and P3 amplitudes were both largest at the FCz sites. Averaged waveforms and topographic maps are shown in Figures 3, 4.

### N2

#### N2 Amplitude

Results revealed that the main effect of age group was not significant, *F*_(1,63)_ = 0.116, *p* = 0.734. The main effect of trial category was not significant, *F*_(1,63)_ = 0.047, *p* = 0.830. The main effect of emotion face type was significant, *F*_(2,126)_ = 6.147, *p* = 0.003, η*_*p*_*^2^ = 0.08, indicating that the N2 evoked by the negative face was larger than the positive and neutral face (*p*s ≤ 0.003), while the difference between positive and neutral face was not significant (*p* = 1.000). The trial category × emotion face type interaction effect was significant, *F*_(2,126)_ = 3.657, *p* = 0.029, η*_*p*_*^2^ = 0.055. Simple effect analysis revealed that, for the Nogo trials, compared with a positive and neutral face, the negative face evoked the largest N2 amplitudes (*p* < 0.001 and *p* = 0.049, respectively); but for Go trials, there was no significant effect (*p* ≥ 0.324). However, there was no significant difference between the Go and Nogo trials for the positive, neutral, and negative face (*p* ≥ 0.062). Other interactions were not significant, *p*s ≤ 0.194.

### P3

#### P3 Amplitude

ANOVA revealed that, the main effect of trial category was not significant, *F*_(1,63)_ = 3.877, *p* = 0.053; and the main effect of age group was not significant, *F*_(1,63)_ = 0.11, *p* = 0.918. The main effect of the emotion face type did reach significance, *F*_(2,126)_ = 6.174, *p* = 0.003, η*_*p*_*^2^ = 0.089, indicating that the P3 amplitudes evoked by negative and positive face were larger than that evoked by neutral face (*p*s ≤ 0.036), but the difference between negative and positive face was not significant (*p* = 1.000). The trial category × emotion face type interaction effect was not significant, *F*_(2,126)_ = 0.773, *p* = 0.464. Other interactions were not significant, *p*s ≥ 0.135.

One-way ANOVA was performed to examine whether there is an effect of P3 evoked by different emotional faces in the Go and Nogo trials, respectively. For Go trials, the main effect of the emotion face type was not significant, *F*_(2,128)_ = 1.544, *p* = 0.218. For Nogo trials, a significant main effect on the emotion face type was observed, *F*_(2,128)_ = 4.095, *p* = 0.019, η*_*p*_*^2^ = 0.060. A *post hoc* test revealed that, compared with the neutral face, the negative face evoked the largest P3 amplitudes (*p* = 0.030), and had no significant difference between the positive and neutral or negative face (*p* = 0.104 and *p* = 1.000, respectively).

### Correlation Analysis

**FIGURE 5:**
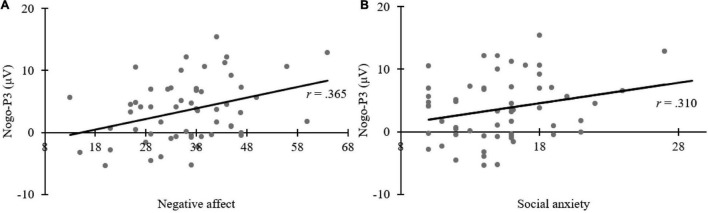
Scatter plots depicting Pearson’s correlation analyses. Panel **(A)** shows the positive correlation between Nogo-P3 amplitudes elicited by viewing neutral faces and negative affect measured by the Early Adolescent Temperament Questionnaire-Revised (EATQ-R). Panel **(B)** shows the positive correlation between Nogo-P3 amplitudes elicited by viewing neutral faces and social anxiety measured by the Social Anxiety Scale For Children (SASC).

The aim was to explore the relationship between ERP and children’s individual factors, Pearson’s correlation analyses were adopted. After controlling for age and gender of children, Pearson’s correlation analyses revealed a significant positive correlation between negative affect and Nogo-P3 elicited by neutral face (*r* = 0.365, *p* = 0.006); and social anxiety was significantly correlated with Nogo-P3 elicited by neutral face (*r* = 0.310, *p* = 0.020), with the higher the social anxiety score, the larger the P3 amplitude. No other correlations were observed (*p*s > 0.05). Correlation results are shown in Figure 5.

## Discussion

This study mainly focused on the AER of 8–12-year-old children, and the implicit emotion Go/Nogo task was adopted. The present study demonstrated that children aged 8–12-year-old could regulate emotion automatically, which is reflected by the Nogo-P3 component. Moreover, the AER ability of children was correlated with the negative effect dimension of temperament and social anxiety. Token together, the findings suggest that 8–12-year-old children can regulate emotion without conscious participation, and temperament affects this ability.

The behavioral results showed that the RT of emotional stimuli in the Go condition was significantly shorter than that of neutral stimuli, which was consistent with previous studies (Zhang and Lu, 2012; Zhang et al., 2016). The accuracy results showed that the accuracy of Go/Nogo tasks for older children was higher than that of younger children, which reflected the enhancement of children’s executive control ability with age. In addition, both the Go and Nogo trials showed an improvement in the accuracy of emotional stimulation (especially positive emotional stimulation), which was closely related to the development level of inhibition and control ability for 8–12-year-old children. Moreover, children’s RT for neutral emotional stimulation in Go trials is long and the accuracy is low, which seems to disrupt the speed-accuracy trade-off, but it is consistent with the research results of significant emotional stimulation (i.e., emotional information is task-relevant information) as materials (Tottenham et al., 2011). Although this study used implicit emotional Go/Nogo tasks (i.e., emotional information is task-irrelevant information), the emotionality of the stimulus is completely accessible to the participants, so children can use the emotionality of the stimulus to guide their responses.

Studies have pointed out that response inhibition and AER accumulate on the Nogo-P3 amplitude (Spronk et al., 2008; Albert et al., 2010). The present study found that the Nogo-P3 amplitude was modulated by emotional valence, with negative emotional faces evoking greater Nogo-P3 amplitudes relative to neutral and positive emotional faces. In the Nogo trials of negative emotional faces, participants processed the negative emotional stimuli more deeply after completing the response inhibition, which caused the participants’ automatic response inhibition to negative emotions, i.e., AER. Response inhibition in the Nogo task was superimposed with AER, automatic emotion inhibition, such that Nogo-P3 had the largest amplitude in Nogo trials for negative emotional faces. This result suggests that 8–12-year-old children could regulate emotion automatically and also suggests that Nogo-P3 can be used as a neural marker of children’s AER.

In the current study, we did not observe any emotion effects of Go-N2, but previous studies suggested that salient stimuli (such as, emotional stimuli) will capture more attention. Therefore the Go-N2 induced by emotional stimuli was higher than that induced by neutral stimuli, indicating Go-N2 reflects automatic top-down attention (Nieuwenhuis et al., 2003; Dennis and Chen, 2007). Since Lamm and Lewis (2010) suggested that the cognitive control of frontal lobe was age-related, one possible explanation was that 8–12-year-old children participated in the current study whose frontal lobe was not mature enough to process emotional information while performing the key press task as adults or adolescents participants. Therefore, the participants focused their attention resources on the main task (key press task), and the effect of emotional stimulation on attention capture decreased, which led to no significant difference in Go-N2 in the frontal lobe.

The Go-P3 reflects motivated attention, and the affective processing bias hypothesis suggests that the amplitude of Go-P3 evoked by emotional stimuli was more positive than neutral stimuli, as the emotional stimuli were processed with a high priority (Eschenbeck et al., 2005; Knyazev et al., 2009). However, in the current study, any emotion effect was not observed during the Go-P3 epoch, which was different from previous studies in adults and adolescents (Zhang and Lu, 2012; Zhang et al., 2016). Zhang et al. (2016) suggest that, because of the limited attentional resources, a decreased Go-N2 would be found before the increased Go-P3 evoked by emotional stimuli. However, in this study, we did not find a decreased Go-N2 before the P3 time window elicited by emotional stimuli, indicating that the automatic control of attention was not evoked. Subsequently, due to the limited attention resources, the resources were unavailable to enhance the Go-P3 amplitude to emotional stimuli.

A large number of previous studies pointed out that Nogo-N2 is an important indicator of cognitive conflict detection, and their findings also found that Nogo-N2 is not modulated by emotional valence, so Nogo-N2 is not directly related to AER (Albert et al., 2011; Zhang and Lu, 2012; Zhang et al., 2016). The results of this study were different from previous studies, and we found that the Nogo-N2 evoked by negative emotional faces was larger than that of neutral and positive emotional faces. According to the characteristics of Nogo-N2, the larger the amplitude, the greater the cognitive conflict experienced by the participants. The results of this study may indicate that the participants experienced greater cognitive conflict in the Nogo trials of negative emotional faces. This may be because in the Nogo trial, after the participants completed response inhibition, the negative emotional stimuli captured the attentional resources as salient stimuli (Lang et al., 1997), which made the participants process them more deeply, so they experienced greater cognitive conflict.

Both the amplitudes of N2 and P3 did not show age difference, which indicates that the AER of 8–12-year-old children is relatively stable, and Ahmed et al. (2015) also found a similar result. However, Zhang et al. (2016) found an age effect in adolescents aged 12–17 years, i.e., older participants had larger ERP amplitude compared with younger participants. Meanwhile, Zhang et al. (2016) found that the RT was faster for older compared with the younger; and the accuracy was higher for older compared with the younger. Our results found that in the Go trials, the participants for the senior group rather than the middle group had higher accuracy. This is closely related to the development level of children and adolescents. Children aged 8–12 years have stable but slow development, so AER is relatively stable; while 12–17 years old are in adolescence, adolescents develop rapidly, and AER changes are more obvious.

The Nogo-P3 is a neural marker of AER, which reflects the process of automatic inhibition of individual responses to emotional stimuli. The results of this study showed that negative affect temperament was positively correlated with Nogo-P3 induced by neutral emotional stimulation. In childhood, the role of temperament is particularly important for children’s emotion regulation competence (Sheese et al., 2009). Children with a high level of negative affect temperament would experience more negative emotions in daily lives, therefore, when facing neutral emotional events, these children might have more negative explanatory bias. There is another possible explanation for the correlation results. In the paradigm used in this study, children need to judge the gender of faces, which undoubtedly calls on children’s face recognition ability. Hills et al. (2011) found that, compared with individuals who experience positive emotion conditions, individual who experienced negative emotion processed face stimuli more elaborately. Consequently, the correlation results in this study might reflect the relationship between temperament and face recognition abilities of children, which means that the more negative emotion experienced, the deeper the processing of face stimuli. In addition, this study also found that social anxiety was positively correlated with Nogo-P3 induced by neutral emotional stimulation, which may be because children with higher scores of social anxiety had poor communication with their peers and often showed withdrawal and avoidance behavior, which affected the individual’s automatic processing of emotional stimuli (Klemanski et al., 2017).

## Limitations and Implications

There are several limitations in the current study. First, this study was a cross-sectional study and failed to fully explain the neurophysiological development of AER in children. Therefore, future studies can use longitudinal data to further investigate the developmental characteristics of children’s AER. Second, our study could not find Go-N2 was an effective indicator of AER in 8–12 years old children. The results may be limited by the experimental paradigm, children in the paradigm need to accurately judge the gender of faces within 250 ms, which may be a little difficult for them. Therefore, future research could develop a paradigm suitable for children of this age. Third, ERP technology has a low spatial resolution which cannot reveal the clear brain mechanism of AER in children. Future research can combine ERP and functional magnetic resonance imaging (fMRI) technology to conduct a deeper investigation of the brain mechanism of children’s AER.

Despite the above limitations, the current study also has some implications. Consistent with prior studies (Yoon et al., 2017), the current study has found that there is a close correlation between children’s emotional regulation and negative emotions (such as, depression and anxiety). In other words, successful emotional regulation plays an important role in children’s mental health. Therefore, for reducing children’s negative emotions, researchers and practitioners should take measures to improve the children’s emotional regulation ability. Moreover, this study found that temperament is an important factor affecting children’s AER, so the training of children’s AER should consider the type of children’s temperament. Combined with previous studies, this finding may indicate that the critical period of AER is in late childhood and adolescence (Zhang et al., 2016). Therefore, we should take into account the impact of critical periods when training individual AER.

## Conclusion

This study aimed to explore children’s AER through a cued-emotion paradigm. The results showed that emotional faces induced greater Nogo-P3 than neutral faces, and Nogo-P3 induced by neutral faces was positively correlated with the negative emotional dimension of temperament and was positively correlated with social anxiety. These results suggest that 8–12-year-old children can regulate emotion automatically, which is reflected in the Nogo-P3 component, and that this ability is closely related to children’s temperament and social anxiety.

## Data Availability Statement

The datasets presented in this study can be found in online repositories. The names of the repository/repositories and accession number(s) can be found in the article/supplementary material.

## Ethics Statement

The studies involving human participants were reviewed and approved by Liaoning Normal University. Written informed consent to participate in this study was provided by the participants’ legal guardian/next of kin.

## Author Contributions

FL and WL contributed to the conception and design of the study, acquisition, analysis, interpretation of the data, and drafting the manuscript. CG and HG contributed to acquisition, analysis, interpretation of the data, and revising the draft critically for important intellectual content. FL, CG, HG, and WL contributed to revising the draft critically for important intellectual content. All authors have approved the final version of the manuscript and agreed to be accountable for all aspects of the study.

## Conflict of Interest

The authors declare that the research was conducted in the absence of any commercial or financial relationships that could be construed as a potential conflict of interest.

## Publisher’s Note

All claims expressed in this article are solely those of the authors and do not necessarily represent those of their affiliated organizations, or those of the publisher, the editors and the reviewers. Any product that may be evaluated in this article, or claim that may be made by its manufacturer, is not guaranteed or endorsed by the publisher.
